# High-dose methotrexate-based chemotherapy for induction remission of newly diagnosed primary CNS lymphoma: A systematic review and meta-analysis

**DOI:** 10.1097/MD.0000000000041363

**Published:** 2025-01-31

**Authors:** Han Shi, Xuefei Sun, Yuchen Wu, Qu Cui, Shengjun Sun, Nan Ji, Yuanbo Liu

**Affiliations:** a Department of Hematology, Beijing Tiantan Hospital, Capital Medical University, Beijing, China.

**Keywords:** combination chemotherapy, cytarabine, high-dose methotrexate, induction remission, primary central nervous system lymphoma, rituximab

## Abstract

**Background:**

This study aimed to comprehensively assess the optimal regimen for high-dose methotrexate (HD-MTX) in treating primary central nervous system lymphoma (PCNSL).

**Methods:**

We have searched 8 databases, including PubMed, EMBASE, Cochrane Library, WOS, Epistemonikos, CNKI, WAN-FANG Database, and CBM, and were selected for the clinical trials about PCNSL. A total of 37 studies were included in our analysis, consisting of 6 randomized controlled trials and 31 single-arm clinical studies.

**Results:**

After analyzing the data from 37 clinical studies, we found that the pooled overall response rate (ORR) for low-dose (<3 g/m^2^), medium-dose (3–5 g/m^2^), and high-dose (>5 g/m^2^) methotrexate (MTX) were 0.78, 0.80, and 0.80, respectively. The pooled 2-year overall survival (OS) for low-dose, medium-dose, and high-dose MTX were 52%, 60%, and 71%, respectively. The ORR, complete response (CR), and 2-year OS of patients who received <5 cycles of MTX were 79%, 41%, and 59%, respectively, whereas those for PCNSL patients who received >5 cycles of MTX were 81%, 54%, and 64%, respectively. The pooled ORR for MTX, dual therapy, triplet therapy, tetrad therapy, and multiple therapy were 71%, 70%, 81%, 85%, and 80%, respectively. The pooled 2-year OS for different numbers of medication combinations were 59%, 52%, 66%, 63%, and 60%, respectively. The addition of cytarabine to MTX-based chemotherapy resulted in higher CR, although no statistically significant difference was observed in OS. Adding rituximab to the treatment regimen improved patients’ progression-free survival without affecting treatment response or OS.

**Conclusion:**

Based on the findings of this study, the treatment strategies of MTX are associated with the prognosis and efficacy response of PCNSL patients. The results suggested that the current recommended HD-MTX dosage of 3.5 g/m^2^ is sufficient for PCNSL to have a favorable treatment response and prognosis. When the number of MTX treatment cycles increases, the therapeutic effect and prognosis of PCNSL patients are improved. The patients treated with MTX-based triplet combination regimens have a better ORR and CR. Although HD-MTX is generally well tolerated, it is necessary to be cautious about the use of multiple therapy that includes cytarabine to prevent potential acute toxicity.

## 1. Introduction

Primary central nervous system lymphoma (PCNSL) is a rare and highly aggressive subtype of non-Hodgkin lymphoma, predominantly localized to the brain parenchyma, spinal cord, cranial nerves, meninges, cerebrospinal fluid, and eyes without systemic dissemination. It comprises 4% of cerebral tumors and 4% to 6% of all extranodal lymphomas, typically presenting as diffuse large B-cell lymphoma (DLBCL) phenotype.^[1]^ The occurrence of PCNSL has exhibited stability or slightly increased in younger immunocompetent individuals. However, there has been a significant rise in the occurrence among adults aged 65 years and above, comprising more than half of all PCNSL patients.^[2]^ Although PCNSL exhibits a relatively positive response to chemotherapy, it presents a refractory and a high recurrent rate.^[3]^ In contrast to systemic DLBCL, this specific subtype demonstrates an unfavorable prognosis, with a 5-year overall survival (OS) rate of only 30%.^[4]^

Due to the existence of the blood–brain barrier (BBB), unique chemotherapy protocols are imperative for PCNSL. A dosage of at least 1 g/m^2^ of high-dose methotrexate (HD-MTX) can penetrate the BBB and achieve therapeutic concentration in brain parenchyma.^[5]^ The current treatment approach for achieving remission of PCNSL is based on a combination of chemotherapy regimens with HD-MTX. Research indicates that the entry of MTX into central nervous system (CNS) is influenced by various factors, including the total dosage, administration rate, dosing interval, and creatinine clearance.^[6]^ Based on the literature reports, the recommended dosage range of HD-MTX in treating PCNSL is 1 to 8 g/m^2^. Some perspectives suggest a minimum HD-MTX dosage of 3 g/m^2^ to achieve an effective concentration in cerebrospinal fluid (CSF) and improve prognosis. The optimal dosage and the infusion method of MTX have not yet been determined. The attainment of a balance between a patient’s drug tolerance and treatment effectiveness is crucial, especially considering the substantial proportion of elderly individuals in the patient population.

Although single-agent MTX treatment can effectively induce remission, it is accompanied by a high rate of substantial incidence of early relapse. The combinations of MTX with cytarabine or rituximab have been demonstrated to enhance chemotherapy’s therapeutic efficacy effectively. With the advancement of molecular biology research in PCNSL, novel therapeutic agents such as Bruton tyrosine kinase inhibitor ibrutinib, PD-1 inhibitor nivolumab, and immunomodulators have gradually emerged.^[7]^ However, different chemotherapy regimens are administered depending on the clinical experience of various medical institutions.^[8]^ As a rare cancer, most clinical studies on PCNSL derivatives are from single-arm clinical trials, lacking randomized controlled trials (RCTs). Therefore, the optimal treatment regimen for PCNSL is still undetermined.

In this article, we performed a systematic review and meta-analysis to explore the optimal MTX administration mode and different combined chemotherapeutical strategies based on MTX for remission induction and prognosis in patients with PCNSL.

## 2. Methods

This meta-analysis was registered in the International Prospective Register of Systematic Reviews (CRD42021265901).

### 2.1. Literature search strategy and data extraction

We conducted a comprehensive search across various foreign databases (PubMed, EMBASE, Cochrane Library, WOS, and Epistemonikos) as well as Chinese databases (CNKI, WANFANG, and CBM) from their respective establishment dates until April 23, 2023. The detailed search strategy can be found in Supplementary material S1, Supplemental Digital Content, http://links.lww.com/MD/O324. In summary, we initially conducted an extensive literature search encompassing PCNSL. Subsequently, we employed reference management software to filter out the literature about MTX. Then, by carefully reviewing titles and abstracts, we identified relevant clinical studies. Finally, after thoroughly reading the full text of selected articles, we determined their suitability for inclusion in this meta-analysis. Two reviewers independently extracted information from the included studies, and the third reviewer oversaw and verified the outcomes of data extraction. In case of disagreements between the 2 reviewers, a consensus was reached through discussion. If disagreements persisted, the third reviewer facilitated further discussion to resolve the issue. In rare cases where no agreement could be reached, a senior researcher was consulted to make the final decision.

### 2.2. Inclusion and exclusion criteria

Currently, there is no standardized approach to treating PCNSL, and the chemotherapy regimens employed by different institutions vary. Additionally, RCTs are scarce. Based on our search results, we incorporated RCTs and phase 2 or higher single-arm clinical trials. Furthermore, including at least 20 patients in each study was mandatory. Moreover, the inclusion criteria for the studies were as follows: (1) patients with newly diagnosed PCNSL; (2) detailed reporting of medication regimen; (3) sufficient outcome data, such as complete response (CR), overall response rate (ORR), and OS; and (4) studies using MTX as first-line treatment. Studies were excluded according to the following criteria: (1) relapsed and refractory PCNSL; (2) secondary CNS lymphoma; (3) CNS prophylaxis of DLBCL; (4) review, case report, animal research, and retrospective study; (5) specialized in intraocular lymphoma, virus-related PCNSL, and PCNSL in children and adolescents.

### 2.3. Quality assessment

Two investigators independently assessed the quality of the included studies. We used the Cochrane Collaboration risk of bias tool to evaluate the included RCTs according to the Cochrane Handbook recommendations. Included single-arm studies were assessed by the methodological index for non-randomized studies.

### 2.4. Statistical analysis

The statistical analyses for single-arm studies were conducted using Stata SE, version 15 (StataCorp, College Station, TX). The metaprop command is utilized to fit both fixed and random effect models for analyzing the effect index. The statistical analyses for RCTs were conducted using Reviewer Manager 5.3. The 95% confidence interval, consisting of an upper and lower limit, was used to represent the effect size of all pooled results. Heterogeneity among the studies was evaluated using the Chi-square test (Q-statistic) and *I*^2^ statistic. If *P* ≥ .10 and/or *I*^2^ < 50%, a fixed effect model (Mantel-Haenszel method) was employed due to low heterogeneity. While a random effect model (Mantel-Haenszel method) was used in cases of significant heterogeneity. The forest plots were generated to present the findings concisely.

## 3. Results

### 3.1. Search results and study selection

**Figure 1.**
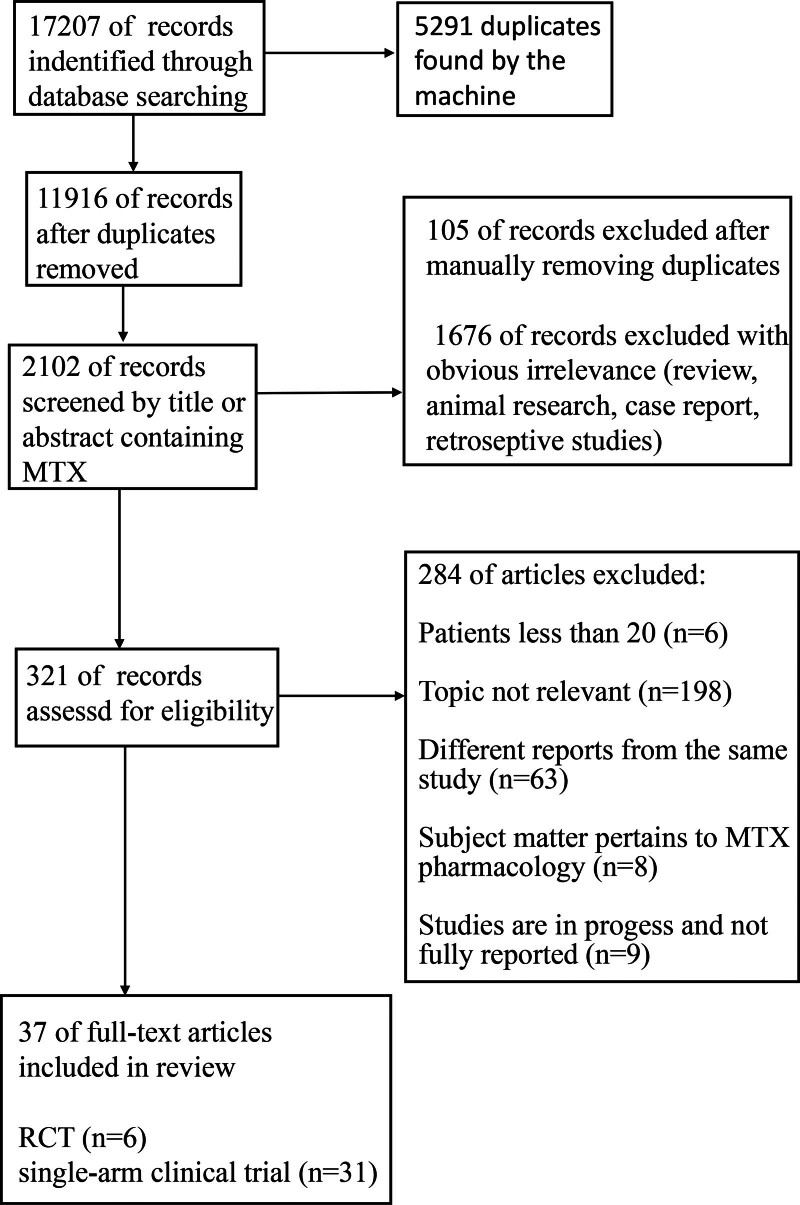
Flowchart of the articles search and screening process.

The flowchart illustrating the selection and inclusion is presented in Figure 1. Following the selection, a total of 37 cohorts were retrieved, consisting of 6 RCTs and 31 single-arm clinical trials in phase 2 or higher.

### 3.2. Study characteristics

We have extracted the following baseline characteristics, including authorship, year of publication, gender distribution, age range, number of patients over 60 years old, Karnofsky performance score, Eastern Cooperative Oncology Group (ECOG) performance status, number of tumors, intraocular involvement, DLBCL phenotype classification, remission induction regimens, single-dose MTX, number of cycles, medication intervals, consolidation therapy regimens. The fundamental features of each study were briefly outlined in Table 1. Due to factors such as death and intolerance to MTX, some patients are considered unsuitable for evaluating treatment response, leading to a discrepancy between initial enrollment patients and individuals utilized for analysis. This analysis comprised a total of 2570 patients. Twenty-three cohorts reported the proportion of elderly patients, with 58% (1049/1796) of patients being over the age of 60 years. Histological classification of PCNSL was reported by 33 cohorts, with DLBCL accounting for the majority (95%) of cases.

**Table 1:** The baseline characteristics of included studies.

Author	Year	N^0^	Male	Age*	Age > 60	ECOG or KPS†	Multifocal sites	Intraocular involvement	DLBCL	Induct- ion	Cycles‡	MTX dosage	Inter-val§	Consoli-dation	N^1^	ORR∥	CR
*Single-arm studies*																	
O’Brien^[1]^	2000	46	33	58 (25–76)	NM	≤1 (22), 2 (18), 3 (6)	19	2/45	UN	MTX	2	1	7	WBRT	39	37	32
Omuro^[2]^	2015	32	17	57 (23–67)	11	80 (40–100)	NM	3	32	RMPV	5–7	3.5	14	ASCT	32	30	14
Pulczynski^[3]^	2015	66	35	64 (40–75)	>65, 27	≤1 (36), 2 (14), 3 (12), 4 (4)	42	4	65	Multiple∥	6	5	21	Chemo	65	59	45
Gerard^[4]^	2011	23	11	60.9 (9.8)	NM	NM	NM	3/15	20	MPV	2	3.5	28	WBRT	22	15	14
Poortmans^[5]^	2003	52	35	51 (21–65)	10	70 (40–100)	21	2/51	NM	MTBMe	2	3	14	WBRT	42	38	17
Pels^[6]^	2003	65	34	62 (27–75)	35	70 (20–90)	NM	1/63	65	Multiple	6	5	14	WBRT	61	43	37
Bessell^[7]^	2001	31	19	59 (21–70)	14	0–2 (19), 3–4 (12)	10	NM	27	CHVD/BVAM	6	1.5	14	WBRT	27	22	18
Adhikari^[8]^	2018	22	13	51.5 (31–67)	NM	1 (4), 2 (5), 3 (13)	NM	5/22	18	RMVP	5	3.5	14	WBRT	19	18	10
Goldkuhl^[9]^	2002	30	18	58 (31–71)	>65, 13	NM	10	0	25	BVDMA	2	6	7	Chemo, WBRT	25	19	9
Illerhaus^[10]^	2016	79	44	56 (51–62)	25	90 (70–90)	54	2	77	RMATh	4	8	10	ASCT	79	73	21
Morris^[11]^	2013	52	30	60 (30–79)	27	70 (50–100)	NM	5	NM	RMPV	5	3.5	14	WBRT	43	41	20
Ghesquières^[12]^	2010	99	51	63 (20–82)	54	0–1 (43), >1 (45)	41/98	6/98	82	MDoVCA	5	1.5 or 3	21	WBRT	82	70	33
Hoang-Xuan^[13]^	2003	50	20	72 (60–81)	50	50 (40–100)	21	6	49	MLPMe	3	1	9	WBRT, chemo	46	24	21
Illerhaus^[14]^	2006	30	25	54 (27–64)	3	70 (30–100)	8	NM	29	MATh	3	8	9	ASCT,WBRT	26	24	10
Ferreri^[15]^	2006	41	NM	NM	NM	NM	NM	NM	NM	MAITh	3	3.5	21	WBRT	41	31	18
DeAngelis^[16]^	2002	98	53	56.5	41	80	NM	1	NM	MVP	5	2.5	14	WBRT, chemo	50	47	29
Abrey^[17]^	2003	28	18	53 (25–71)	NM	70 (30–100)	NM	3	NM	MA	5	3.5	14	ASCT	25	16	
Glass^[18]^	2016	53	25	57 (24–73)	20	NM	NM	NM	48	RMTe	5	3.5	14	WBRT, chemo	35	30	18
Swinnen^[19]^	2018	25	10	57 (51–68)	10	0–1 (16), 2–3 (9)	NM	NM	NM	Multiple	5	3.5	14	NM	24	20	16
Batchelor^[20]^	2003	25	17	59.8 (12.2)	NM	78.4 (13.1)	14/24	5	22	MTX	8	8	14	WBRT	23	17	12
Laack^[21]^	2011	36	19	60.5 (34–69)	NM	0–1 (30), 2–3 (5)	NM	0	34	CHVD/BVAM	6	1.5	14	WBRT	36	20	10
Rubenstein^[22]^	2013	44	21	61 (12–76)	NM	0–1 (13), 2–3 (23), 4–5 (4)	NM	1	43	RMTe	8	8	14	Chemo	44	34	29
Fritsch^[23]^	2017	107	57	73 (66–85)	NM	70 (30–100)	65	1	107	RMPL	9	3	14	Chemo	65	53	38
Chamberlain^[24]^	2010	40	25	61.5 (18–93)	>70, 17	80 (50–100)	12	NM	40	RM	4–6	8	14	Chemo	40	32	24
Shah^[25]^	2007	30	17	57 (30–76)	NM	70 (50–90)	NM	3	NM	RMPV	7	3.5	14	WBRT	27	25	21
Herrlinger^[26]^	2002	37	NM	60 (37–73)	21	70 (50–100)	14/37	3/28	35	MTX	6	8	14	Chemo	28	13	11
Montemurro^[27]^	2006	23	12	55 (18–70)	NM	70 (30–100)	12	0	22	MTX	2	8	10	ASCT	16	14	3
Illerhaus^[28]^	2008	30	15	70 (57–79)	>65, 27	60 (30–90)	11	1	30	MPL	9	3	14	WBRT	27	19	12
Korfel^[29]^	2005	56	NM	60(41–76)	NM	NM	NM	NM	52	MBPD	3	1.5	28	WBRT	50	39	30
Abrey^[30]^	2000	52	31	65(27–89)	NM	70 (30–100)	NM	10/19	36	MPV	5	3.5	7	WBRT	48	43	27
Colombat^[31]^	2006	25	9	51(21–60)	NM	0–2 (17), 3–4 (8)	15	NM	25	MVBP	2	3	21	ASCT	25	21	11
*RCTs*																	
Bromberg^[32]^	2019	99	48	61 (55–67)	52	0–1 (73), 2–3 (26)	46	8	97	RMVBP	2	3	28	WBRT, chemo	99	85	30
Bromberg^[32]^	2019	100	61	61 (56–66)	53	0–1 (71), 2–3 (29)	51	3	99	MVBP	2	3	28	WBRT, chemo	100	86	36
Ferreri^[33]^	2016	75	46	58 (50–64)	NM	>1, 27	45	5	75	MA	4	3.5	21	WBRT, ASCT	75	40	17
Ferreri^[33]^	2016	69	44	57 (53–63)	NM	>1, 23	40	1	69	RMA	4	3.5	21	WBRT, ASCT	69	51	21
Ferreri^[33]^	2016	75	46	57 (53–62)	NM	>1, 24	41	1	75	RMATh	4	3.5	21	WBRT, ASCT	75	65	37
Ferreri^[34]^	2009	39	NM	59 (25–74)	NM	>1, 14	21	4	34	MA	4	3.5	21	WBRT	39	27	18
Ferreri^[34]^	2009	40	NM	58 (27–72)	NM	>1, 20	25	5	35	MTX	4	3.5	21	WBRT	40	16	7
Omuro^[35]^	2015	47	26	72 (60–84)	NM	60 (40–90)	26	9	45	MPVA	3	3.5	28	Chemo	45	37	28
Omuro^[35]^	2015	48	25	73 (60–85)	NM	70 (40–100)	28	10	45	MTe	3	3.5	28	Chemo	42	30	19
Thiel^[36]^	2010	526	UN	UN	337	UN	UN	UN	UN	MIf	6	4	14	WBRT, chemo	430	283	182
Wu^[37]^	2018	25	16	57 (16–67)	NM	>1, 15	22	NM	25	MA	4	3.5	21	WBRT	25	21	10

A, cytarabine; B, carmustine; chemo, chemotherapy; CR, complete rate; C, cyclophosphamide; D, dexamethasone; Do, doxorubicin; F, fotemustine; I, idarubicin; If, ifosfamide; L, lomustine; H, doxorubicin; NM, not mentioned; M, methotrexate; Me, methylprednisolone; ORR, overall response rate; P, procarbazine; R, rituximab; T, teniposide; Th, thiotepa; Te, temozolomide; UN, uncertainty; V, vincristine; N^0^, the total number of patients in the cohort; N^1^, numbers that meet the criteria for response evaluation.

KPS = Karnofsky performance score, RCTs = randomized controlled trials, WBRT = whole-brain radiation therapy.

* Median (range) or mean (SD).

† Number of individuals with varying ratings in parentheses (ECOG), KPS is represented as median (range).

‡ Number of MTX cycles.

§ MTX administration interval time.

∥ ≥5 agents.

### 3.3. MTX dose and treatment response in PCNSL patients

**Figure 2.**
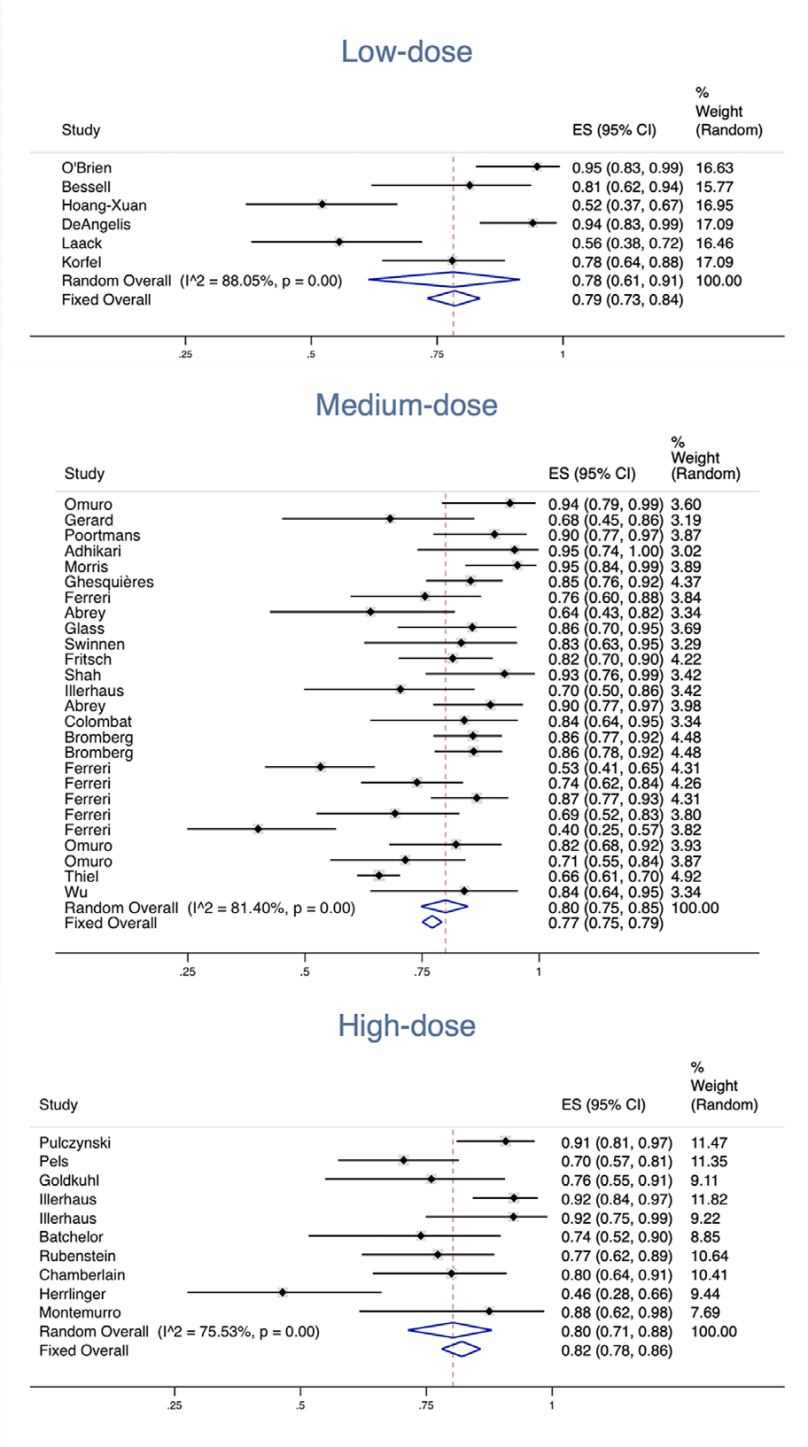
Overall response rates of different MTX dosages. MTX = methotrexate.

The dosage of MTX employed in the meta-analysis ranged from 1 to 8 g/m^2^. According to the varying doses of MTX administered, we categorized all included studies into 3 distinct groups. The low-dose group (<3 g/m²) was reported in all 6 studies,^[9–14]^ while the medium-dose group (3–5 g/m²) was reported in 21 studies^[15–35]^ and the high-dose group (>5 g/m²) was reported in 10 studies.^[36–45]^ In the low-dose group, the pooled ORR in 6 studies was 0.78 (95% CI, 0.61–0.91), with an *I*^2^ value of 88.05%. The pooled CR in 6 studies was 0.57 (95% CI, 0.42–0.71), with an *I*^2^ value of 81.76%. In the medium-dose group, the pooled ORR in 26 studies was 0.80 (95% CI, 0.75–0.85), with an *I*^2^ value of 81.40%; the pooled CR in 25 studies was 0.45 (95% CI, 0.40–0.50), with an *I*^2^ value of 70.80% (a literature did not record CR). In the high-dose group, the pooled ORR in 10 studies was 0.80 (95% CI, 0.71–0.88), with an *I*^2^ value of 75.53%; the pooled CR in 10 studies was 0.47 (95% CI, 0.36–0.59), with an *I*^2^ value of 81.26% (Fig. 2).

### 3.4. MTX cycles and treatment response in PCNSL patients

**Figure 3.**
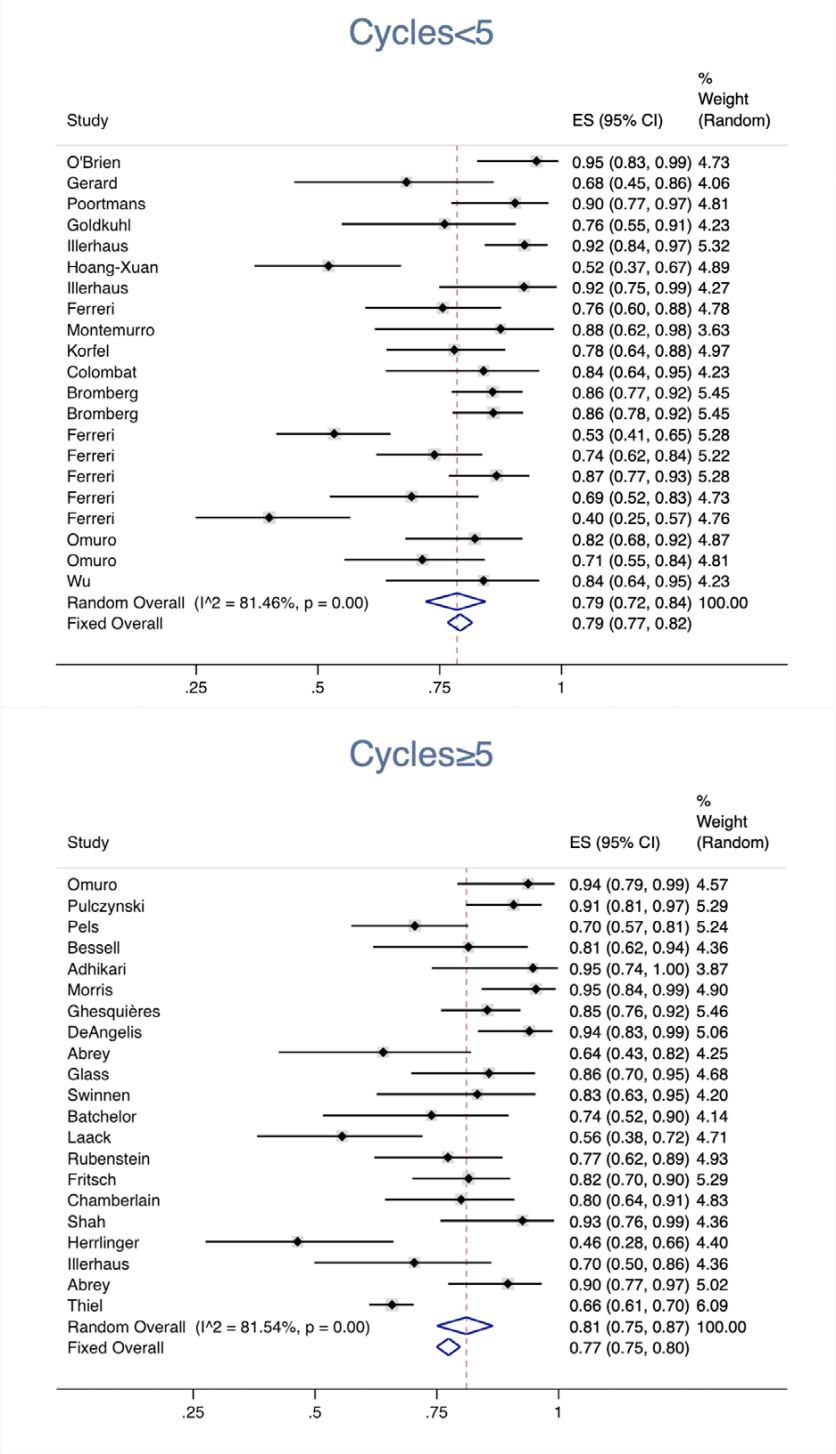
Overall response rates for different cycles of HD-MTX. HD-MTX = high-dose methotrexate.

Thirty-seven studies were included in this analysis. Two-thirds of the studies utilized medication intervals of 2-three weeks, and some employed weekly intervals. Medication intervals of 4 weeks were typically observed when administering multiple (≥5 agents) medications. The number of MTX cycles varied between 2 and 8. Based on the different cycles of MTX administered, we categorized all included studies into 2 distinct groups: low cycles (2–4) were reported in 14 studies,^[9,14,16,17,21,29–33,35,38,39,44]^ and high cycles (5–8) were reported in 23 studies.^[10–13,15,18–27,34,36–38,40–43,45]^ In the low-cycle group, the pooled ORR in 21 studies was 0.79 (95% CI, 0.72–0.84), with an *I*^2^ value of 81.46%. The pooled CR in 21 studies was 0.41 (95% CI, 0.35–0.48), with an *I*^2^ value of 78.62%. In the high-cycles group, the pooled ORR in 21 studies was 0.81 (95% CI, 0.75–0.87), with an *I*^2^ value of 81.54%; the pooled CR in 20 studies was 0.54 (95% CI, 0.48–0.59), with an *I*^2^ value of 68.20% (Fig. 3).

### 3.5. MTX-based combination drugs and treatment response in PCNSL patients

**Figure 4.**
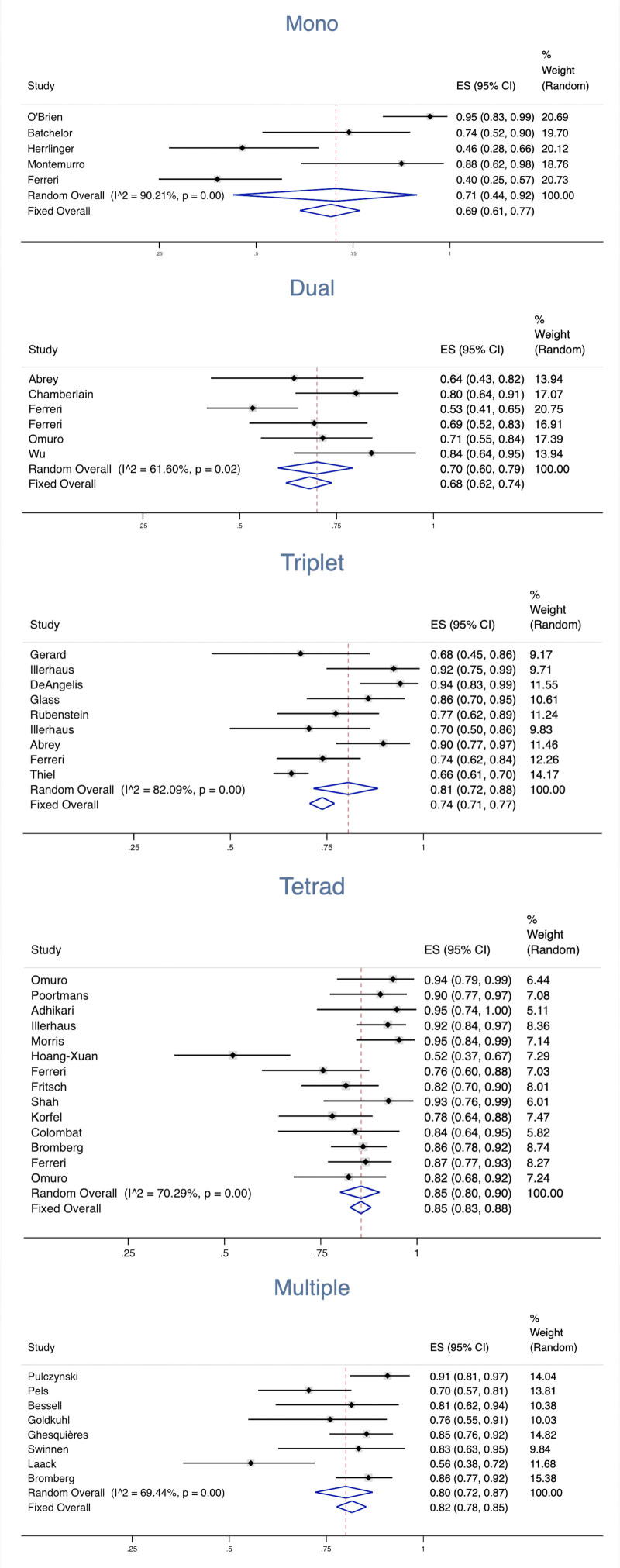
Overall response rates of MTX-based combination regimens with different numbers of agents. MTX = methotrexate.

Thirty-seven studies were eligible for the analysis of the combination of different numbers of drugs based on MTX. The results showed that the pooled ORR for MTX in 5 studies was 0.71 (95% CI, 0.44–0.92), with an *I*^2^ value of 90.21%. The pooled CR for MTX in the 5 studies was 0.42 (95% CI, 0.17–0.69), with an *I*^2^ value of 90.73%. The pooled ORR for dual therapy in 6 studies was 0.70 (95% CI, 0.60–0.79), with an *I*^2^ value of 61.6%. The pooled CR for dual therapy in the 5 studies was 0.42 (95% CI, 0.28–0.56), with an *I*^2^ value of 77.50%. The pooled ORR for triplet therapy in 9 studies was 0.81 (95% CI 0.72, 0.88), with an *I*^2^ value of 82.09%. The pooled CR for triplet therapy in the 9 studies was 0.49 (95% CI, 0.41–0.57), with an *I*^2^ value of 67.61%. The pooled ORR for tetrad therapy in 14 studies was 0.85 (95% CI 0.80, 0.90), with an *I*^2^ value of 70.29%. The pooled CR for tetrad therapy in the 14 studies was 0.48 (95% CI, 0.41–0.55), with an *I*^2^ value of 69.11%. The pooled ORR for multiple therapy in 8 studies was 0.80 (95% CI, 0.72–0.87), with an *I*^2^ value of 69.44%. The pooled CR for multiple treatments in 8 studies was 0.49 (95% CI, 0.37–0.62), with an *I*^2^ value of 84.49% (Fig. 4).

### 3.6. Overall survival

We analyzed the effects of MTX doses, cycles, and combinations on the OS of PCNSL patients. The pooled 2-year OS for low-dose MTX was 0.52 (95% CI, 0.40–0.64), with an *I*^2^ value of 77.44%; the pooled 2-year OS for medium-dose MTX was 0.60 (95% CI, 0.55, 0.65), with an *I*^2^ value of 74.54%; the pooled 2-year OS for high-dose MTX was 0.71 (95% CI, 0.62, 0.79), with an *I*^2^ value of 71.13%. Considering MTX cycles, the pooled 2-year OS for low-cycles of MTX was 0.59 (95% CI, 0.52–0.66), with an *I*^2^ value of 80.72%; the pooled 2-year OS for high-cycles of MTX was 0.64 (95% CI, 0.58, ‐0.69), with an *I*^2^ value of 73.12%. In addition, the effect of MTX-based combination drugs on the OS of patients with PCNSL was analyzed. The results showed that the pooled 2-year OS for MTX was 0.59 (95% CI, 0.45–0.73), with an *I*^2^ value of 63%. The pooled 2-year OS for dual therapy in 5 studies was 0.52 (95% CI, 0.42–0.63), with an *I*^2^ value of 62.14%. The pooled 2-year OS for triplet therapy in 9 studies was 0.66 (95% CI, 0.58–0.74), with an *I*^2^ value of 74.39%. The pooled 2-year OS for tetrad therapy was 0.63 (95% CI 0.54–0.72), with an *I*^2^ value of 84.99%. The pooled 2-year OS for multiple therapy was 0.60 (95% CI, 0.52–0.68), with an *I*^2^ value of 67.12%. The pooled 2-year OS of MTX-based different chemotherapy regimens were summarized in Table 2.

**Table 2:** Two-year OS for various treatment modalities.

Pooled 2-year OS (95% CI)		
Different doses of MTX	Low-dose	0.52 [0.40, 0.64]
	Medium-dose	0.60 [0.55, 0.65]
	High-dose	0.71 [0.62, 0.79]
Different cycles of MTX	Cycles 2–4	0.59 [0.52, 0.66]
	Cycles 5–8	0.64 [0.58, 0.69]
MTX-based combination regimens with different numbers of agents	Mono	0.59 [0.45, 0.73]
	Dual	0.52 [0.42, 0.63]
	Triplet	0.66 [0.58, 0.74]
	Tetrad	0.63 [0.54, 0.72]
	Multiple	0.60 [0.52, 0.68]

### 3.7. Treatment with or without rituximab

**Figure 5.**
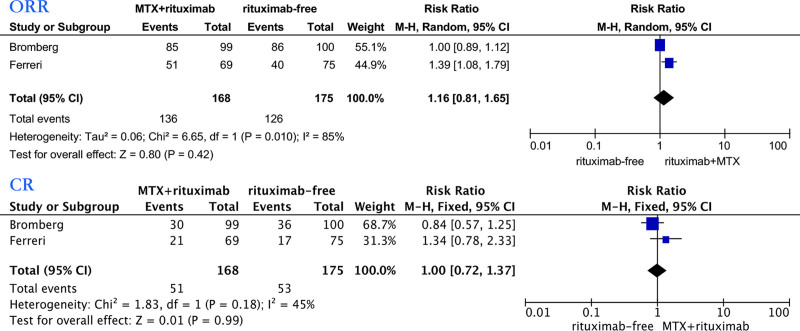
Forest plot of the ORR/CR for treatment with the rituximab group versus rituximab-free group. CR = complete response, ORR = overall response rate.

**Figure 6.**
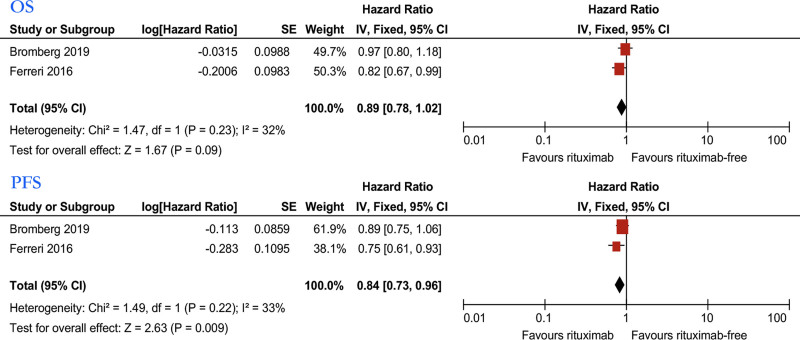
Forest plot of the OS/PFS for treatment with the rituximab group versus rituximab-free group. OS = overall survival, PFS = progression free survival.

We first analyzed the relationship between MTX combined with rituximab or rituximab and the efficacy of PCNSL patients in 2 studies^[30,31]^ (Fig. 5). Based on RCTs, we used the Mantel–Haenszel random model to analyze patients’ ORR. The results showed that rituximab was not statistically related to ORR (relative risk [RR]: 1.16; 95% CI, 0.81–1.65). We also used the Mantel–Haenszel fixed model for CR analysis as low heterogeneity (*I*^2^ = 46%); the results showed that rituximab was not statistically related to CR (RR: 1.00, 95% CI, 0.72–1.37).We further analyzed the impact of MTX combined with rituximab or rituximab on the OS of PCNSL patients (Fig. 6). Pooled data from 2 studies showed no significant difference in the OS (hazard ratio [HR]: 0.89, 95% CI, 0.78–1.02), with no considerable heterogeneity (*I*^2^ = 32%). Pooled data showed that the rituximab group was associated with better progression-free survival (PFS) (HR: 0.84, 95% CI, 0.73–0.96), with no significant heterogeneity (*I*^2^ = 33%).

### 3.8. Treatment with or without cytarabine

**Figure 7.**
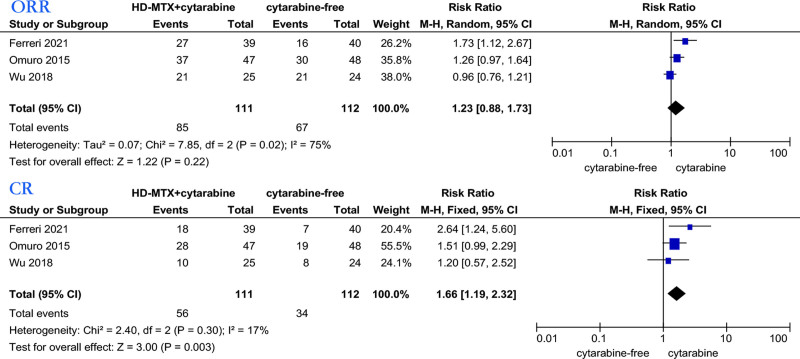
Forest plot of the ORR/CR for treatment with the cytarabine versus cytarabine-free group. CR = complete response, ORR = overall response rate.

**Figure 8.**
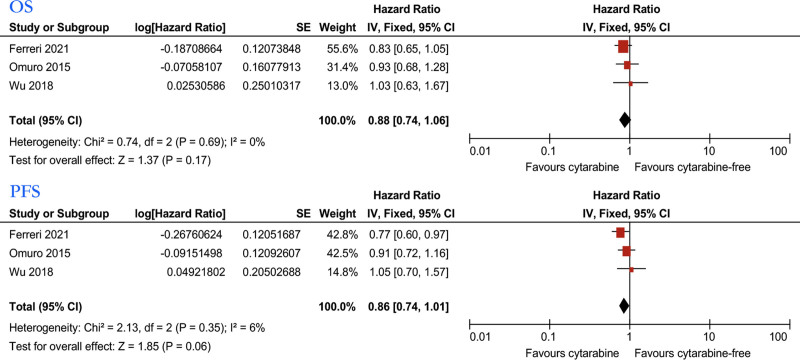
Forest plot of the OS/PFS for treatment with the cytarabine versus cytarabine-free group. OS = overall survival, PFS = progression free survival.

Similarly, we analyzed the relationship between MTX combined with cytarabine or cytarabine and treatment response (Fig. 7) and prognosis (Fig. 8) of PCNSL patients in 3 studies.^[32,33,35]^ We employed the Mantel–Haenszel random-effects model to analyze patients’ ORR. The findings indicated that cytarabine demonstrated no statistically significant association with ORR (RR: 1.23, 95% CI, 0.88–1.73). For CR analysis, we employed the Mantel–Haenszel fixed-effects model. The results favored the cytarabine group (RR: 1.66, 95% CI, 1.19–2.32). Pooled data from 3 studies showed no significant difference in the OS (HR: 0.88, 95% CI, 0.74–1.06), with low heterogeneity (*I*^2^ = 0%). Pooled data showed no significant difference in the PFS (HR: 0.86, 95% CI, 0.74–1.01), with no significant heterogeneity (*I*^2^ = 6%). Pooled data from 3 studies showed no significant difference in the OS (HR: 0.88, 95% CI: 0.74, 1.06), with low heterogeneity (*I*^2^ = 0%). Pooled data showed no significant difference in the PFS (HR: 0.86, 95% CI, 0.74–1.01), with no significant heterogeneity (*I*^2^ = 6%).

### 3.9. Chemotherapy-related acute toxicity

In the meta-analysis, all studies recorded data related to chemotherapy-associated deaths, a total of 138 cases (5%) of mortality among the 2570 patients. Pulczynski et al^[36]^ reported that all treatment-related deaths occurred following the initial administration of cytarabine, with the primary cases attributed to multi-organ failure resulting from neutropenia and sepsis-induced. The results of Pels et al^[37]^ discovered that in 5 deceased patients, there were 3 experienced fatalities after cycles of cytarabine-based treatment, while the other 2 patients used cytarabine in the initial course of therapy. Two studies reported a mortality rate of approximately 17%, and these studies involved combination therapy, with cytarabine being one component.

The detail in reporting acute toxicity data varied among different studies, and we found that patients exhibit well tolerance to chemotherapy. The most common acute toxicity was hematopoietic suppression. In 10 studies, over half of patients experienced grade 3 to 4 neutropenia. Three of the studies utilized multiple chemotherapies. In contrast, the other 7 regimens were RMATh, MATh, MAITh, MA, and RMA (M, MTX; R, rituximab; A, cytarabine; Th, thiotepa; I, ifosfamide). The occurrence of grade 3 to 4 thrombocytopenia is similar to that of neutropenia. Grade 3 to 4 anemia was infrequent in nearly all of the studies. Forty-eight of 50 patients experienced anemia in MLPMe (MTX, lomustine, procarbazine, and methylprednisolone) regimens, and 98 of 107 patients experienced anemia in RMPL (MTX, procarbazine, lomustine, and rituximab) regimens. Other acute toxicities are less frequent, and their severity is lower. The incidence of grade 3 to 4 renal toxicity was found to be <8% in 17 cohorts. The incidence of grade 3 to 4 hepatotoxicity and grade 3 to 4 infection ranges from 2% to 60% and 3% to 44% observed in 17 cohorts. Grade 3 to 4 infection was observed in 17 cohorts, with an incidence ranging from 3% to 44%. Grade 3 to 4 thrombosis/embolism was observed in 10 cohorts, with an incidence ranging from 2% to 9%. In addition, other significant acute toxicities were also observed, including stomatitis and nausea, while fortuitous occurrences included peripheral neuropathy, allergy, encephalopathy, and arrhythmias.

## 4. Discussion

This systematic review and meta-analysis included RCTs and single-arm clinical trials, focusing primarily on exploring the optimal regimen for HD-MTX in treating primary PCNSL. As our study primarily analyzed the effect of the MTX regimen on remission induction and prognosis, we deemed treatment response rate as the primary outcome endpoint. In certain single-arm studies where PFS was unavailable as an outcome indicator, we used 2-year OS for prognostic observation.

The therapeutic dosage of MTX is established by identifying the minimum effective dose required to produce cytotoxic effects in CSF and brain parenchyma. It is uncertain which metric can enhance treatment responsiveness and prognosis in PCNSL patients while ensuring optimal patient tolerance. The current dosage range of MTX for PCNSL is 1 to 8 g/m^2^. The recommended dosage of MTX for combination chemotherapy is 3.5 to 4.5 g/m^2^. Some experts suggested intravenous administration of MTX at a minimum dose of 3 g/m^2^ within 2 to 3 hours to ensure effective penetration through the BBB and generate therapeutic concentration in CSF. Several studies employed a 1 g/m^2^ dosage, possibly for safety considerations, which can penetrate brain parenchyma. Meanwhile, studies report increasing MTX dosage to 8 to improve patient prognosis. This study showed that the pooled ORR for MTX at a <3.5 g/m^2^ dose was 0.78, with a pooled CR of 0.57. In the medium-dose group, the ORR and CR for dose 3.5 to 5 g/m^2^ were 0.80 and 0.45, respectively. While the dose >5 g/m^2^ was 0.80 and 0.47 in the high-dose group. The pooled 2-year OS of patients in low-dose, medium-dose, and high-dose MTX were 52%, 60%, and 71%, respectively. These findings suggest that an increased dosage of MTX beyond the effective dose is unnecessary for improving treatment response rates of PCNSL patients. However, increasing the dosage of MTX can have a positive effect on improving patient prognosis.

This meta-analysis sheds light on the relationship between MTX cycles and treatment outcomes in patients with PCNSL. The pooled ORR and CR for MTX treatment cycles 2 to 4 and 5 to 8 were 0.79 and 0.41, 0.81 and 0.54. This meta-analysis highlights the association between MTX treatment cycles and clinical outcomes in patients with PCNSL. The pooled ORR and CR for MTX regimens with 2 to 4 cycles were 0.79 and 0.41, respectively, while those with 5 to 8 cycles achieved 0.81 and 0.54. These findings suggest that both low- and high-cycle MTX regimens demonstrate favorable ORRs, though high-cycle regimens are associated with superior CR rates and more consistent outcomes. The results emphasize the need for personalized MTX treatment strategies. While higher-cycle regimens appear to enhance CR rates, the decision to increase the number of cycles should be guided by the patient’s tolerance and overall health status. Future randomized controlled trials are necessary to confirm these findings and provide more definitive recommendations on the optimal number of cycles and dosing intervals. In addition, this analysis provides valuable insights into the effects of MTX doses, treatment cycles, and combination regimens on the OS of patients with PCNSL. The findings highlight that both the intensity of MTX dosing and the number of treatment cycles have a meaningful impact on 2-year OS, with higher doses and more cycles generally associated with improved survival outcomes. These research findings suggested that increasing the number of MTX treatment cycles had little effect on the overall rate and patient survival. Consistent with the results of Curry et al, the average cumulative dose of MTX did not significantly affect patients.^[46]^

The treatment response of patients for the MTX monotherapy showed similar results with the MTX-based dual regimen. For the triplet regimens, the results exhibited a higher response rate with an ORR and CR of 0.81 and 0.49. The patients had a better ORR and CR of 0.85 and 0.48 in the tetrad regimens. In conclusion, as the number of combined drugs increases, treatment response has a noticeable upward trajectory. The pooled 2-year OS of patients in monotherapy, dual therapy, triplet therapy, tetrad therapy, and multiple therapy were 59%, 52%, 66%, 63%, and 60%, respectively. There is no significant correlation between the number of combination drugs and prognosis. The prognosis heavily depends on the efficacy of individual drugs in the combination therapy. The prognosis results may depend on the effectiveness of particular drugs in the combination therapy.

The first RCT assessing cytarabine conducted by the International Extranodal Lymphoma Study Group (IELSG) demonstrated an increase in failure-free survival with MTX + cytarabine compared to MTX alone.^[32]^ The study found that high-dose HTX combined with high-dose cytarabine can significantly improve patients’ CR. However, the results of the pooled effect estimation indicate that there is no significant effect on OS/PFS. Given the limited availability of RCTs, the accuracy of the results for cytarabine may be influenced by variations in the specific medication regimens used in each study. We conducted further exploration, and the results indicate that. Cytarabine was more favorable for PFS after removing the RCT of Wu et al comparing fotemustine + teniposide + dexamethasone to MTX + cyclabaine.^[35]^ Rituximab is a monoclonal antibody specifically targeting the CD20 cell surface protein, significantly improving the efficacy of systemic DLBCL treatment. In PCNSL, pooled CR and OS show no significant correlation with rituximab. On the other hand, rituximab can significantly improve patients’ PFS. In the HOVON 105/ALLG NHL 24 trial, the results failed to demonstrate significant improvement in PFS with the addition of rituximab. However, the subgroup analysis results from Bromberg et al indicate that improved PFS with rituximab in patients aged up to 60 years may be due to younger patients receiving more whole-brain radiation therapy, which disrupts the BBB and enhances the permeability of rituximab into the brain.^[30]^ In the International Extranodal Lymphoma Study Group 32 trial, the combination treatment regimen incorporating rituximab to the MTX + cytarabine significantly improves both CR (HR, 0.69, 95% CI 0.54–0.88) and PFS (HR, 0.52, 95% CI 0.32–0.86) in patients. In this meta-analysis, the results did not demonstrate the effectiveness of rituximab in treatment response in PCNSL patients. It could be attributed to the use of various combination chemotherapies in the HOVON 105/ALLG NHL 24 trial, leading to a lack of overlap with the benefits of rituximab, ultimately affecting the outcomes. Rituximab appears to confer a beneficial impact in the context of mono or dual MTX therapy. A previous meta-analysis demonstrated that adding rituximab improved outcomes, including CR, 2-year PFS, and 2-year OS in both RCT and retrospective studies. However, it is worth noting that 2 different types of research may lead to bias in the results.^[47]^

As is well known, patients generally exhibit good tolerance to chemotherapy, with bone marrow suppression being the most common side effect. In the study, the mortality rate related to chemotherapy is 5%, and the leading cause of death is treatment-related infectious complications due to bone marrow suppression. After analyzing the toxic reactions in the article, it could be inferred that age is not the determining factor for the occurrence of severe toxic side effects among patients. This could be due to lower MTX doses in some early patients, and most studies lack data on the age of patients experiencing toxic reactions. The tolerability of MTX monotherapy in elderly patients was acceptable. For monotherapy regimens, there were no statistically significant differences in the toxic side effects between patients >60 years and those <60 years old. However, considering the potential toxicity of combination therapy in elderly patients, caution is advised in medication use. The results of DeAngelis et al^[12]^ found that 46% of patients <60 years old and 64% of patients >60 years old experienced grade 3 to 4 toxicity with triplet therapy. Fritsch et al found that 87% of early patients with tetrad therapy experienced at least one ⩾grade 3 toxicity, while the percentage was 71.1% for those treated with triplet therapy.^[25]^ It is noteworthy that neutropenia is the most common occurrence in multiple therapies involving cytarabine. Results from 2 studies indicated that the addition of cytarabine in combination therapy resulted in treatment-related death. Besides, Ferreri et al demonstrated that Grade 3 to 4 hematological toxicity was a more common in the MTX combined with the cytarabine group than in the MTX group (92% vs 15%).^[32]^ The combination of cytarabine and MTX may cause synergistic myelosuppression in PCNSL. This study has certain limitations, mainly due to the heterogeneity in treatment approaches across different studies. First, maintaining coherence in other aspects proves challenging while classifying based on a single trait. In this article, we have only provided information on the cycles and dosage of HD-MTX. However, some patients may experience a reduction in MTX dosage or changes in the treatment cycles during therapy. Second, the prognosis is influenced by multiple factors, including age and ECOG/Karnofsky performance score performance status. However, obtaining comprehensive patient-level data for analysis is difficult. When analyzing the effect of MTX on patient prognosis, it is necessary to consider the potential impact of consolidation therapy on prognosis. Thirdly, due to the limitations of the prognosis data from each study, we only focus on 2-year OS. Fourthly, the level of detail in reporting acute toxicity varied among different studies. We only conducted a descriptive analysis of the acute toxicity. Furthermore, for analyzing the effect of cytarabine or rituximab on treatment response and prognosis of patients, lacking qualified RCTs and heterogeneity in chemotherapy regimens result in the pooled results being overly influenced by individual studies. In future research, we will focus on collecting and analyzing more comprehensive data to conduct in-depth studies that address these limitations.

## 5. Conclusions

The study suggests that the recommended mainstream dose of MTX at 3.5 g/m^2^ is sufficient to achieve desirable response rates. With an increase in the number of MTX cycles, there is an improvement in the treatment efficacy and prognosis for PCNSL patients. Compared to MTX monotherapy, the patients treated with triple therapy have a better ORR and CR. However, the addition of medications based on the triple therapy did not significantly improve treatment response. No significant association exists between the number of agents utilized in combined chemotherapy and prognosis. Although the addition of cytarabine or rituximab has shown favorable results in the PFS of patients, their efficacy as part of multiple therapies is still limited. The MTX-based combination chemotherapy is well tolerated in PCNSL patients, and elderly patients can also safely receive MTX with reduced dosage and preventative measures. Furthermore, to avoid potential acute toxicity, it is necessary to be cautious about the use of multiple therapy that includes cytarabine.

## Author contributions

**Conceptualization:** Xuefei Sun, Yuanbo Liu.

**Data curation:** Han Shi, Qu Cui.

**Validation:** Yuchen Wu.

**Visualization:** Yuchen Wu, Shengjun Sun, Nan Ji.

**Writing – original draft:** Han Shi.

**Writing – review & editing:** Han Shi.

## Supplementary Material


