# Supplementary material for: High-dose methotrexate-based chemotherapy for induction remission of newly diagnosed primary CNS lymphoma: A systematic review and meta-analysis
Source: Medicine (Baltimore). 2025 Jan 31;104(5):e41363. doi: 10.1097/MD.0000000000041363 (PMC11789877; doi:10.1097/MD.0000000000041363)
Supplement: Supplementary file 1 [file medi-104-e41363-s001.docx]

**The detailed search strategy**

**PubMed（n=3821）**

1. "primary central nervous system lymphoma"[Title/Abstract]
2. "PCNSL"[Title/Abstract]
3. "primary CNS lymphoma"[Title/Abstract]
4. "diffuse large b cell lymphoma"[Title/Abstract]
5. "Lymphoma, Large B-Cell, Diffuse"[Mesh]
6. "DLBCL"[Title/Abstract]
7. "central nervous system"[Title/Abstract]
8. "CNS"[Title/Abstract]
9. #4 OR #5 OR #6
10. #7 OR #8
11. #9 AND #10
12. OR/#1-#3
13. #11 OR #12

**EMBASE（n=3967）**

1. ‘primary central nervous system lymphoma’/exp
2. ‘diffuse large b cell lymphoma’/exp
3. ‘central nervous system’/exp
4. ‘primary central nervous system lymphoma’:ab,kw,ti
5. ‘pcnsl’:ab,kw,ti
6. ‘primary CNS lymphoma’:ab,kw,ti
7. ‘diffuse large b cell lymphoma’:ab,kw,ti
8. ‘dlbcl’:ab,kw,ti
9. ‘central nervous system’:ab,kw,ti
10. ‘cns’:ab,kw,ti
11. #2 OR #7 OR #8
12. #3 OR #9 OR #10
13. #11 AND #12
14. #1 OR #4 OR #5 OR#6 OR #13
15. #14 AND [medline]/lim
16. #14 NOT #15

**Cochrane Library（n=435）**

1. MeSH descriptor: [Lymphoma, Large B-Cell, Diffuse] explode all trees
2. MeSH descriptor: [Central Nervous System] explode all trees
3. 'diffuse large b cell lymphoma':ti,ab,kw
4. 'dlbcl':ti,ab,kw
5. 'central nervous system':ti,ab,kw
6. 'cns':ti,ab,kw
7. #1 OR #3 OR #4
8. #2 OR #5 OR #6
9. #7 AND #8
10. 'primary central nervous system lymphoma':ti,ab,kw
11. 'pcnsl':ti,ab,kw
12. 'primary CNS lymphoma':ti,ab,kw
13. #10 OR #11 OR #12
14. #9 OR #13

**Web of Science（n=5413）**

1. Topic: (“CNS”)
2. Topic: (“central nervous system”)
3. Topic: (“DLBCL”)
4. Topic: TS=(“diffuse large b cell lymphoma”)
5. Topic: (“primary central nervous system lymphoma”)
6. Topic: (“PCNSL”)
7. Topic: (“primary CNS lymphoma”)
8. #1 OR #2
9. #3 OR #4
10. #8 AND #9
11. #5 OR #6 OR #7
12. #10 OR #11

**Epistemonikos（n=99）**

1. title/abstract:("DLBCL")
2. title/abstract:("diffuse large b cell lymphoma")
3. title/abstract:("CNS")
4. title/abstract:("central nervous system")
5. #1 OR #2
6. #3 OR #4
7. #5 AND #6
8. title/abstract:("primary central nervous system lymphoma")
9. title/abstract:("PCNSL")
10. title/abstract:("primary CNS lymphoma")
11. OR/#8-#10
12. #7 OR #11

**CNKI（n=1371）**

1. “原发性中枢神经系统淋巴瘤”[主题]
2. “原发中枢神经系统淋巴瘤”[主题]
3. “PCNSL”[主题]
4. “中枢神经系统弥漫大B细胞淋巴瘤”[主题]
5. OR/#1-#4

**万方（n=1019）**

1. “原发性中枢神经系统淋巴瘤”[主题]
2. “原发中枢神经系统淋巴瘤”[主题]
3. “PCNSL”[主题]
4. “中枢神经系统弥漫大B细胞淋巴瘤”[主题]
5. OR/#1-#4

**CBM（n=910）**

1. "原发性中枢神经系统淋巴瘤"[常用字段:智能]
2. "原发中枢神经系统淋巴瘤"[常用字段:智能]
3. "PCNSL"[常用字段:智能]
4. "中枢神经系统弥漫大B细胞淋巴瘤"[常用字段:智能]
5. OR/#1-#4
